# Use of Remdesivir in children with COVID-19: report of an Italian multicenter study

**DOI:** 10.1186/s13052-024-01606-z

**Published:** 2024-02-27

**Authors:** Lorenza Romani, Marco Roversi, Stefania Bernardi, Elisabetta Venturini, Silvia Garazzino, Daniele Donà, Andrzej Krzysztofiak, Carlotta Montagnani, Elisa Funiciello, Francesca Ippolita Calò Carducci, Caterina Marabotto, Elio Castagnola, Filippo Salvini, Laura Lancella, Andrea Lo Vecchio, Luisa Galli, Guido Castelli Gattinara, Italian Society of Pediatric Infectious Diseases (SITIP)

**Affiliations:** 1 Infectious Diseases Unit, Bambino Gesù Children’s Hospital, IRCCS, Rome, Italy; 2 PhD Course “Immunology, Molecular Medicine and Applied Biotechnology”, University of Rome Tor Vergata, Rome, Italy; 3 Infectious Diseases Unit, Meyer Children’s University Hospital, Florence, Italy; 4 Pediatric Infectious Diseases Unit, Regina Margherita Children’s Hospital, University of Turin, Turin, Italy; 5 Division of Pediatric Infectious Diseases, Department for Woman and Child Health, University of Padua, Padua, Italy; 6 Department of Pediatrics, Bambino Gesù Children’s Hospital, IRCCS, Rome, Italy; 7 Hematology and Oncology, Department of Pediatrics, IRCCS Istituto Giannina Gaslini, Genoa, Italy; 8 Pediatrics Division, Azienda Sanitaria Territoriale Grande Ospedale Metropolitano Niguarda, Milan, Italy; 9 Department of Translational Medical Sciences, Federico II University, Naples, Italy; 10 Institute of Child Health, Bambino Gesù Children’s Hospital, IRCCS, Rome, Italy

**Keywords:** COVID-19, Remdesivir, Pneumonia

## Abstract

**Background:**

COVID-19 is generally milder in children than in adults, however severe infection has been described in some patients. Few data are available on use of Remdesivir (RDV) in children, as most clinical trials focused on adult patients. We report a multicenter study conducted in 10 Italian Hospitals to investigate the safety of RDV in children affected by COVID-19.

**Methods:**

We collected the clinical data of children with COVID-19 treated with RDV between March 2020 and February 2022 in 10 Italian hospitals. Clinical data were compared according to a duration of RDV therapy more or less than 5 days. Linear regression model was used to determine the association of significant variables from the bivariate analysis to the duration of RDV therapy.

**Results:**

A total of 50 patients were included, with a median age of 12.8 years. Many patients had at least one comorbidity (78%), mostly obesity. Symptoms were fever (88%), cough (74%) and dyspnea (68%). Most patients were diagnosed with pneumonia of either viral and/or bacterial etiology. Blood test showed leukopenia in 66% and increased C-reactive protein (CRP) levels in 63% of cases. Thirty-six patients received RDV for 5 days, nine patients up to 10 days. Most children who received RDV longer were admitted to the PICU (67%). Treatment with RDV was well tolerated with rare side effects: bradycardia was recorded in 6% of cases, solved in less than 24 h after discontinuation. A mild elevation of transaminases was observed in 26% of cases, however for the 8%, it was still detected before the RDV administration. Therefore, in these cases, we could not establish if it was caused by COVID-19, RDV o both. Patients who received RDV for more than 5 days waited longer for its administration after pneumonia diagnosis. The presence of comorbidities and the duration of O2 administration significantly correlated with the duration of RDV therapy at the linear regression analysis.

**Conclusion:**

Our experience indicates that RDV against SARS-CoV-2 is safe and well-tolerated in pediatric populations at high risk of developing severe COVID-19. Our data suggest that delaying RDV therapy after diagnosis of pneumonia may be associated with a longer duration of antiviral therapy, especially in patients with comorbidities.

## Background

SARS-CoV-2 infection predominantly occurs in children with less severe clinical aspects than in adults, requiring hospitalization only in 5–10% of cases [1–4]. The majority of children with COVID-19 is asymptomatic or has mild symptoms, most commonly fever, cough, pharyngitis, gastrointestinal symptoms, and changes in sense of smell or taste [5–8]. Although the course of COVID-19 disease is generally mild, cases of severe infection have been described in a small proportion of children: in these, therapeutic options such as dexamethasone, antivirals or convalescent plasma may be considered [9, 10]. To date, the optimal therapy for children with severe COVID-19 is unknown, but expert guidance suggested the use of antiviral therapy with Remdesivir (RDV) [9, 11]. RDV has received emergency approval for treating COVID-19, although few data in children are available and most clinical trials have focused on adult patients [12, 13]. RDV is a nucleotide analog that inhibits RNA-dependent RNA polymerase of several viruses, including SARS-CoV-2 [14]. In May 2020, Goldman et al. published the results of the SIMPLE study, showing that a 10-day course of RDV was superior to placebo in reducing recovery time in hospitalized adults with severe COVID-19 and that the 5-day and the 10-day-course were similar in terms of outcomes [15].

To date, RDV is recommended for the treatment of severe COVID-19 in hospitalized children, based on adult data and studies that demonstrated a shorter time to clinical recovery, especially in those requiring supplemental oxygen, without the need for mechanical ventilation [16, 17]. Recently, on November 2022, the European Medicines Agency and the Italian Medicines Agency extended the approval of treatment for COVID-19 with RDV to pediatric patients aged 28 days or older and weighing ≥ 3 kg with positive SARS-CoV-2 viral test results, whether hospitalized or not, with mild-to-moderate COVID-19 and at high risk of progression to severe disease, including hospitalization or death [18, 19].

To date, few studies and case reports have described the use of RDV in children [20–23].

We hereby report the results of a nationwide multicenter study promoted by the Italian Society of Pediatric Infectious Diseases to investigate the safety and tolerability of RDV in children and the clinical characteristics of treated pediatric patients.

## Methods

We retrospectively analyzed the clinical data of 50 pediatric patients with COVID-19 treated with RDV between March 2020 and February 2022 in 10 Italian hospitals, listed here: Bambino Gesù Children’s Hospital (Rome), Meyer Children’s University Hospital (Florence), Regina Margherita Children’s Hospital (Turin), University Hospital of Padua (Padua), IRCCS Istituto Giannina Gaslini Hospital (Genoa), “Azienda Sanitaria Territoriale Grande Ospedale Metropolitano Niguarda” Hospital (Milan), “SS. Antonio e Biagio e Cesare Arrigo” (Alessandria) Children’s Hospital, “ASST Spedali Civili” (Brescia) Hospital, S. Croce e Carle Hospital (Como), Infermi Hospital (Rimini), ASST Valle Olona Hospital of Busto Arsizio (Varese).

All subjects under 18 years with documented SARS-CoV-2 infection treated with RDV were included. Diagnosis of infection was established with the presence of at least one respiratory specimen positive for SARS-CoV-2 nucleic acid using a validated Real-Time reverse-transcriptase Polymerase-Chain-Reaction (RT-PCR) assay. Molecular tests analyzed the envelope protein gene (E), the nucleocapsid protein gene (N) and the RNA-dependent RNA polymerase gene (RdRp) and all fulfilled performance criteria established by the European Commission [24]. RDV was administered at a dose of 5 mg/kg on day 1 and 2.5 mg/kg from day 2 in children less than 40 kg, at a dose of 200 mg on the first day and 100 mg from the second day in those weighing more than 40 kg. Liver enzymes were monitored every 2 to 3 days in all patients who received the drug. Renal function was monitored during and after treatment. COVID-19-related complications were defined as follows: (a) clinical and/or radiological pneumonia; (b) severe acute respiratory illness (SpO2 < 92% associated with tachypnea and/or other signs of respiratory failure); (c) acute respiratory distress syndrome; (d) neurological disturbances; (e) dehydration requiring intravenous rehydration; (f) severe bacterial supra-infection; (g) specific involvement of a single organ/apparatus requiring hospitalization (i.e., myocarditis, pericarditis, pancreatitis, etc.); (h) multisystem inflammatory syndrome temporarily related to COVID-19 (MIS-C) according to CDC criteria [25].

The following clinical data were collected from each patient’s medical records: sex; weight; age; hospitalization; comorbidities; concomitant therapies; reported contact with SARS-CoV-2 infected individual; symptoms and complications of COVID-19; type and length of oxygen (O2) therapy; clinical outcome (full recovery without sequelae or death); laboratory workup, including transaminase and creatinine levels; dose, length and timing of RDV administration; side effects.

All variables were compared between patients undergoing RDV therapy for more than 5 days or for 5 days or less, following the same classification adopted in the study by Goldman et al. conducted on adult patients [15]. We identified clinically and statistically significant variables through bivariate analysis. These variables then served as independent variables in both linear regression analyses. In the linear regression, we adopted the days of RDV therapy as the continuous dependent variable.

### Statistical analysis

Statistical analysis was performed with IBM SPSS Statistics software version 23.0 (IBM Corp. Armonk, NY). A two-tailed *p* value < 0.05 was set for statistical significance. All continuous variables were expressed as means with standard deviations (SDs) or medians with interquartile ranges (IQRs), and compared by Student’s t test or Mann-Whitney’s U test according to the data distribution. All categorical variables were expressed as absolute numbers and percentages (taking into account missing data) and compared by chi-square test and Fisher’s exact test, as appropriate.

### Ethical approval

The study protocol was approved by Bambino Gesù Children’s Hospital Ethical Committee on 26-03-2022 (protocol number 336). Patients were included after providing written informed consent from their parents/caregivers. This study was undertaken in accordance with Good Clinical Practice guidelines and the Declaration of Helsinki.

## Results

A total of 50 patients were included in the study. Of these, 12 were admitted to the Bambino Gesù Children’s Hospital (Rome), 10 to the Meyer Children’s University Hospital (Florence), 9 to the Regina Margherita Children’s Hospital (Turin), 8 to the University Hospital of Padua (Padua), 3 to the IRCCS Istituto Giannina Gaslini (Genoa), 3 to the hospital “Azienda Sanitaria Territoriale Grande Ospedale Metropolitano Niguarda” (Milan), 1 to the children’s hospital “SS. Antonio e Biagio e Cesare Arrigo” (Alessandria), 1 to the hospital “ASST Spedali Civili” (Brescia), 1 to the S. Croce e Carle Hospital (Como), 1 to the hospital “Ospedale degli Infermi” of Rimini (Rimini) and 1 the ASST Valle Olona hospital of Busto Arsizio (Varese).

The clinical characteristics of the patients are detailed in Table 1.

**Table 1:** Characteristics of study sample

Total	50	NA
Males - n (%)	32 (64)	0
Weight (kg) - median ± IQR (range)	65.5 ± 47.5 (3.2–130)	9
Age (years) - median ± IQR (range)	12.8 ± 5.2 (0.02–17.5)	2
Hospitalization (days) - median ± IQR (range) PICU admission - n (%) PICU admission (days)	12 ± 14 (2–63) 16 (34) 15 ± 18 (2–27)	6 3 2
Comorbidities - n (%) Obesity Neurological disorder Immunodeficiency Pulmonary or thoracic condition Congenital disorder Other	39 (78) 11 (28) 9 (23) 8 (21) 3 (8) 4 (10) 4 (10)	0
Concomitant therapies - n (%)	13 (30)	6
Symptoms - n (%)		0
Fever Temperature (°C, max value) - median ± IQR (range) Cough Dyspnea Rhinorrhea Sore throat Chest pain Diarrhea Abdominal pain Vomiting Fatigue Anorexia Headache Joint/muscle pain Dysgeusia/anosmia Hypotonia Rash Seizures Conjunctivitis	44 (88) 39 ± 1.0 (36.0–40.8) 37 (74) 34 (68) 10 (20) 11 (22) 7 (14) 7 (14) 6 (12) 6 (12) 5 (10) 4 (8) 4 (8) 3 (6) 2 (4) 2 (4) 2 (4) 2 (4) 1 (2)	- 4 - - - - - - - - - - - - - - - - -
Complications - n (%) Pneumonia Viral Bacterial Mixed Acute respiratory failure ARDS MIS-C	32 (76) 48 (96) 25 (63) 2 (5) 13 (33) 29 (58) 10 (21) 3 (6)	8 0 - - - 0 2 0
Administration of O2 - n (%) Mask/nasal cannulae Mask/nasal cannulae (days)** High-flow nasal cannulae - HFNC High-flow nasal cannulae - HFNC (days)** Non-invasive ventilation - NIV Non-invasive ventilation - NIV (days)** Mechanical ventilation Mechanical ventilation (days)** Extra-Corporeal Membrane Oxygenation - ECMO Extra-Corporeal Membrane Oxygenation - ECMO** Total duration of O2 administration/ventilation (days)**	38 (78) 31 (63) 4 ± 3 15 (38) 6 ± 8 (1–25) 12 (25) 4.5 ± 6 (1–14) 7 (14) 16 ± 14 (3–23) 2 (5) 9.5 ± / (2–17) 3 ± 7 (0–27)	1 1 10 11 4 2 2 1 2 11 0 1
Laboratory workup		
Leukocytosis - n (%) Leukopenia - n (%) Increased PCR - n (%) Increased PCT - n (%) Bacterial coinfection - n (%)	8 (18) 27 (66) 31(63) 6 (15) 9 (22)	6 9 1 10 9
Clinical outcome		
Recovery without sequelae - n (%) Death - n (%)	34 (89) 2 (4)	12 ?

*Summary measures were calculated accounting for missing values

**Expressed as median ± IQR (range)

Most patients were males (*n* = 32, 64%). The median age was 12.8 years (range 0.02–17.5). Among them, only one was a neonate. Most of the patients included in the study had at least one comorbidity (78%), mainly obesity (28%) and 8 children (16%) had more than a comorbidity. The symptoms at the clinical presentation of SARS-CoV-2 infection and during the admission were fever (88%), cough (74%), dyspnea (68%), sore throat (22%) and rhinorrhea (20%). Forty-eight patients (96%) were diagnosed with pneumonia of viral (63%), bacterial (5%) and viral plus bacterial (33%) etiology (based on serology and culture analysis). When necessary (78%), oxygen was administered via a nasal mask/cannula (63%), high-flow nasal cannulae (38%), noninvasive ventilation (25%) or mechanical ventilation (14%). Two patients required extracorporeal membrane oxygenation (ECMO), one affected by Adenosine deaminase deficiency (ADA deficiency) and autoimmune vasculitis, the other one with sickle cell disease. None of the patients recruited in the study had any viral respiratory co-infection while bacterial coinfection was reported in nine children (18%), mainly accompanied by bacteremia. Three patients received the diagnosis of MIS-C. In these cases RDV was prescribed because of the evidence of SARS-CoV-2 nasal swab positive and pneumonia. Laboratory workup showed leukopenia in 66% and increased C-reactive protein (CRP) levels in 63% of cases. The median time from symptoms onset to RDV administration was 6 days (range 0–15), while the median time from diagnosis of pneumonia to RDV administration was 2 days (range 0–8). Thirty-two patients (68%) received RDV for 5 days, nine patients (22%) for more than 5 days (max 10 days). For several patients the length of treatments was not reported. The decision to extend the treatment was based on clinical judgment and the disease severity (34% were admitted to PICU). Before RDV administration, the median maximum aspartate amino-transferase (AST) value was 40 U/l (range 9–280) and the median maximum alanine amino-transferase (ALT) value was 34 U/l (range 6–302). After RDV administration, the median maximum AST value was 43 U/l (range 19–164) and the median maximum ALT value was 57 U/l (range 6–350). Of these, thirteen children (26%) had an AST value between 50 and 200 U/l, but four of them (8%) had an AST value above 100 U/l already before RDV treatment (data not shown). Sixteen patients (32%) had a maximum ALT value after RDV administration between 50 and 350 U/l, but four patients (8%) had an ALT value above 200 U/l already before RDV treatment. Before and after RDV administration, we found a statistically significant difference in AST values, but not ALT. (*p* = 0.019 vs. *p* = 0.955, data not shown on Table 2). None of the patients recorded an increase in creatinine value. The comparison of median creatinine values before and after RDV administration proved no significant difference (*p* = 0.550, data not shown on Table 1). Bradycardia was reported in 6% of cases; for all of these, RDV therapy was discontinued. Two children died, both with severe comorbidities, namely gangliosidosis and ADA deficiency. RDV administration, safety measures after RDV administration and other therapies are reported in Table 2.

**Table 2:** RDV administration, safety measures and other therapies

Total	50	NA	
RDV administration			
Dose of RDV Duration of therapy (days) - median ± IQR (range) ≤ 5 days - n (%) > 5 days - n (%) Time from symptoms onset to administration (days) - median ± IQR (range) Time from pneumonia to administration (days) - median ± IQR (range)	4 ± 1 (1–10) 32 (78) 9 (22) 6 ± 3 (0–15) 2 ± 2 (0–8)	9 9 - - 9 10	
Safety evaluation of RDV administration			
AST pre-RDV (U/L, max value)– median ± IQR (range) AST post-RDV (U/L, max value)– median ± IQR (range) ALT pre-RDV (U/L, max value) - median ± IQR (range) ALT post-RDV (U/L, max value) - median ± IQR (range) Creatinine pre-RDV (mg/dl, max value) - median ± IQR (range) Creatinine post-RDV (mg/dl, max value) - median ± IQR (range) eGFR (ml/min, max value)* - median ± IQR (range) Ipertransaminasemia - n (%) Bradicardia - n (%) Rash - n (%) Renal insufficiency (according to eGFR) - n (%)	40 ± 47 (9–280) 43 ± 47 (19–164) 34 ± 70 (6–302) 54 ± 108 (6–350) 0.6 ± 0.3 (0.1–0.9) 0.6 ± 0.4 (0.1–0.9) 120 ± 91 (79–236) 14 (44) 2 (4) 3 (6) 0 (0)	13 18 13 18 13 16 21 18 0 0 21	
Antibiotic therapy			
Macrolides - n (%) Azithromycin - n (%) Clarithromycin - n (%) Duration (days) - median ± IQR (range)	10 (24) 6 (15) 3 (7) 6.5 ± 5 (0–13)	8 9 9 -	
Other than macrolides** - n (%)			
Ceftriaxone or other cephalosporin - n (%) Piperacillin-tazobactam or tazobactam - n (%) Amoxicillin-clavulanate or other beta-lactam - n (%) Teicoplanin - n (%) Aminoglycosides - n (%) Fluoroquinolones - n (%) Linezolid - n (%) Meropenem - n (%) Metronidazole - n (%) Trimethoprim-sulfamethoxazole - n (%) Duration (days) - median ± IQR (range)	13 (34) 12 (32) 7 (18) 4 (11) 3 (8) 3 (8) 1 (3) 1 (3) 1 (3) 1 (3) 9 ± 11 (4–28)	12 12 12 12 12 12 12 12 12 12 -	
Corticosteroid therapy			
Duration (days) - median ± IQR (range) Desametasone - n (%) Methylprednisolone - n (%) Prednisone - n (%)	9.5 ± 9 (3–36) 9 (27) 19 (58) 3 (9)	12 17 17 17	
Other therapies			
Heparin - n (%) Duration (days) - median ± IQR (range) Monoclonal antibody - n (%) Duration (days) - median ± IQR (range) Hyperimmune plasma - n (%)	20 (63) 11 ± 20 (2–58) 3 (7) 2.5 ± / (1–4) 2 (8)	18 2 8 1 25	

*The lowest value of the eGFR calculated by the MDRD, CDK-EPI or Cockroft-Gault formulas was reported for children aged 8 or more years

**Cefotaxime (*n* = 1), ceftazidime (*n* = 1); amoxicillin (*n* = 1), ampicillin (*n* = 1), ampicillin-sulbactam (*n* = 1); amikacin (*n* = 2), gentamicin (*n* = 1); ciprofloxacin (*n* = 2), levofloxacin (*n* = 1)

All variables were compared between patients undergoing RDV therapy for more than 5 days or for 5 days or less. This bivariate analysis is reported in Table 3.

**Table 3:** Comparison of patients based on days of RDV therapy

	RDV therapy > 5 days	RDV therapy ≤ 5 days	*p*-value
**Total**	**9**	**32**	**-**
Males - n (%)	7 (78)	19 (59)	0.445
Weight (kg) - median ± IQR (range)	36.0 ± 42.5 (3.2–70.0)	67.5 ± 45.3 (4.0–130)	**0.022**
Age (years) - median ± IQR (range)	11.6 ± 13.1 (0.02–17.5)	12.8 ± 4.4 (0.2–17.1)	0.415
Hospitalization (days) - median ± IQR (range)	20.5 ± 24 (10–42)	10 ± 13 (2–63)	**0.034**
PICU admission - n (%) PICU admission (days)	6 (67) 22 ± 7.3 (15–24)	7 (22) 7 ± 9 (4–15)	**0.018** **0.010**
Comorbidities - n (%) Obesity Neurological disorder Primary or secondary immunodeficiency Pulmonary or thoracic condition Congenital disorder Other	6 (67) 0 (0) 2 (22) 2 (22) 0 (0) 0 (0) 0 (0)	27 (84) 9 (28) 5 (16) 5 (16) 2 (6) 4 (13) 3 (9)	0.342 0.167 0.637 0.637 1.000 0.559 1.000
Concomitant therapies - n (%)	2 (25)	8 (30)	1.000
Reported contact with SARS-CoV-2 infected (%)	6 (67)	15 (47)	0.454
Symptoms - n (%)			
Fever Temperature (°C, max value) - mean ± SD (range)	8 (89) 39.1 ± 1.0 (38.0–40.8)	30 (94) 38.9 ± 0.5 (37.5–40.0)	0.535 0.317
Cough Dyspnea Rhinorrhea Sore throat Chest pain Diarrhea Abdominal pain Vomiting Fatigue Anorexia Headache Joint/muscle pain Dysgeusia/anosmia Hypotonia Rash Seizures Conjunctivitis	6 (67) 7 (78) 5 (56) 0 (0) 0 (0) 3 (38) 2 (22) 2 (22) 0 (0) 2 (20) 0 (0) 0 (0) 0 (0) 0 (0) 1 (11) 0 (0) 0 (0)	25 (78) 22 (69) 4 (13) 8 (25) 5 (16) 4 (13) 3 (9) 3 (9) 5 (16) 2 (7) 3 (9) 3 (9) 2 (6) 1 (3) 1 (3) 1 (3) 0 (0)	0.662 0.702 **0.014** 0.164 0.563 0.128 0.299 0.299 0.568 0.213 1.000 1.000 1.000 1.000 0.395 1.000 -
Complications - n (%) Pneumoniae Viral Bacterial Mixed Acute respiratory failure ARDS MIS-C	7 (100) 9 (100) 3 (43) 0 (0) 4 (57) 7 (78) 5 (56) 2 (22)	20 (77) 31 (97) 18 (69) 1 (4) 6 (23) 16 (50) 2 (7) 1 (3)	0.301 1.000 0.337 1.000 0.161 0.254 **0.003** 0.116
Administration of O2 - n (%)			
Mask/nasal cannulae High-flow nasal cannulae - HFNC Non-invasive ventilation - NIV Mechanical ventilation Extra-Corporeal Membrane Oxygenation - ECMO	5 (56) 2 (29) 3 (38) 4 (44) 0 (0)	21 (66) 9 (38) 7 (22) 1 (3) 0 (0)	0.701 1.000 0.388 **0.007** -
Total duration of O2 administration/ventilation (days)**	5 ± 15.5 (0–24)	2.5 ± 6 (0–19)	0.392
Clinical outcome			
Recovery without sequelae - n (%) Death - n (%)	5 (71) 0 (0)	26 (96) 1 (3)	0.101 1.000
Laboratory workup			
Leukocytosis - n (%) Leukopenia - n (%) Increased PCR - n (%) Increased PCT - n (%) Bacterial coinfection - n (%)	2 (22) 7 (78) 6 (67) 3 (33) 4 (67)	6 (17) 14 (61) 21 (68) 2 (7) 24 (86)	0.653 0.441 1.000 0.070 0.281
RDV administration			
Time from onset to RDV administration (days)**	8 ± 2 (5–11)	5.5 ± 3 (0–15)	0.069
Time from pneumonia to RDV administration (days)**	3 ± 2 (2–8)	1 ± 2 (0–7)	**0.010**
Safety evaluation of RDV administration			
AST (U/L, max value) - median ± IQR (range)	43 ± 26 (22–62)	43 ± 72 (19–164)	0.584
ALT (U/L, max value) - median ± IQR (range)	25 ± 30 (21–58)	66 ± 123 (6–350)	0.057
Creatinine (mg/dl, max value) - median ± IQR (range)	0.4 ± 0.4 (0.2–0.8)	0.6 ± 0.3 (0.1–0.9)	0.259
Ipertransaminasemia - n (%) Bradicardia - n (%) Rash - n (%)	0 (0) 0 (0) 0 (0)	15 (65) 2 (6) 3 (9)	**0.028** 1.000 1.000
Antibiotic therapy			
Macrolides - n (%) Duration (days) - median ± IQR (range)	3 (33) - -	6 (22) 6.5 ± 8 (0–9)	0.660 0.480
Other than macrolides - n (%) Duration (days) - median ± IQR (range)	7 (78) 9 ± 16 (5–28)	22 (69) 9.5 ± 9 (4–23)	0.702 0.836
Corticosteroid therapy - n (%) Duration (days) - median ± IQR (range)	7 (78) 11 ± 24 (4–36)	27 (84) 9 ± 7 (3–36)	0.637 0.784
Heparin - n (%) Duration (days) - median ± IQR (range)	3 (43) 24 ± 13 (8–28)	9 (38) 8.5 ± 11 (2–58)	1.000 0.940

*Summary measures were calculated accounting for missing values

**Expressed as median ± IQR (range)

Only 41 patients had complete data on the duration of RDV therapy. Of these, 32 (78%) were administered RDV for more than 5 days and 9 (22%) for 5 days or less. The comparison showed a more frequent admission to the pediatric intensive care unit (PICU) in patients treated with RDV for more than 5 days than in children treated for 5 days or less (67% vs. 22%, *p* = 0.010). Acute respiratory distress syndrome (ARDS) was also more frequent in the former (56% vs. 7%, *p* = 0.003). Not surprisingly, patients treated with RDV for longer also more frequently required mechanical ventilation (44% vs. 3%, *p* = 0.007). Interestingly, patients who were given RDV for more than 5 days also waited longer for its administration after the detection of COVID-19-associated pneumonia (3 vs. 1 median days, *p* = 0.010). Regarding the safety of RDV therapy, no differences were found between the two groups in terms of the frequency and degree of adverse events.

The linear regression analysis showed that the presence of comorbidities (*p* = 0.027) and the duration of O2 administration (*p* = 0.020) significantly correlated with the duration of RDV therapy (Table 4). Male sex also approached significance in the correlation with RDV therapy (*p* = 0.052).

**Table 4:** Linear regression analysis (dependent variable: duration of RDV therapy in days)

Variable	Beta	C.I. 95%		*p*-value
		Lower bound	Upper bound	
Sex (male)	1.313	-0.009	2.635	0.052
Duration of O2 administration (days)	0.122	0.021	0.224	**0.020**
Time from pneumonia to RDV administration (days)	0.336	-0.047	0.719	0.083
Comorbidity (yes)	1.793	0.219	3.367	**0.027**

## Discussion

We report data from a cohort of children with severe COVID-19 disease treated with RDV. To date, only one US retrospective study of 77 pediatric patients with SARS-CoV-2 treated with RDV reported good tolerance to the drug with a low incidence of serious adverse events (16%) [20]. In Spain, a nationwide multicenter observational study of 8 children with confirmed SARS-CoV-2 infection treated with RDV did not observed RDV-related adverse outcomes [21]. This is therefore the second largest dataset on the use of RDV for the treatment of pediatric COVID-19: in this cohort, the majority of children were treated with RDV because they had a severe form of COVID-19 and serious comorbidities, as widely reported in literature [10, 26]. In fact, children with comorbidities, including cardiac disease, neurologic disorders, diabetes, obesity (particularly severe obesity), chronic lung disease, feeding tube dependence, malignancies, sickle cell anemia, and immunocompromised status are often associated to severe form of COVID-19 [2, 27–32].

Recently, obesity has been recognized as a significant risk factor for COVID-19 related prognosis. Brambilla et al. described how obesity increases the risk of hospitalization, intensive care unit admission, need for mechanical ventilation, and death among children and adolescents with COVID-19 [19]. Indeed, the chronic inflammation, nutritional, cardiac, respiratory, and immunological impairment characterizing obesity contribute to the risk of severe COVID-19 [19]. The second common comorbidity in our cohort was neurological disorder. These data are in line with pediatric epidemiologic studies of SARS-CoV-2 infection and are consistent with those reported by Centers for Disease Control and Prevention surveillance data on children hospitalized with COVID-19, highlighting the potential role of these conditions in predisposing patients to severe illness [26].

Expert groups recommend the use of RDV mainly in children with “severe COVID-19” defined as a new or increased demand for supplemental oxygen compared with baseline [16, 17, 33]. However, in our study we administered RDV also in a proportion of children diagnosed with severe pneumonia before the actual need for supplemental oxygen, with few side effects. Therefore, this can be considered as an additional criterion for the use of RDV in children with severe COVID-19.

Overall, RDV was well tolerated in our cohort. Therapy was discontinued in 3 cases because of bradycardia, a side effect also reported by Eleftheriou et al. in 3 of 4 children treated with RDV. After drug discontinuation, the heart rate was back to normal within 24 h [22]. Therefore, we suggest to perform an ECG monitoring before and during RDV treatment in order to highlight this adverse effect as soon as possible. Regarding the mild elevation of liver transaminases observed in our study (47%), it is not possible to define if it is attributable to COVID-19 disease, RDV, or both, as 1/3 of patients showed an increase before treatment. It is also interesting to note that only the increase of AST values before and after RDV administration showed a moderate statistical significant (*p* = 0.019). A recent review on the use of RDV in children reported elevation of transaminases in 32.1% of cases [34]. However, the highest level of ALT and AST observed in our population was 350 U/L e 164 U/L respectively. Furthermore, we found a statistically significant difference, with higher incidence of elevation of transaminases in patients treated with RDV for 5 or less days, suggesting the role of agents other than RDV in the occurrence of this finding. However, the presence of high level of transaminases may have influenced the choice of a more restrained use of the drug, thus no more than 5 days of standard treatment.

Recently, interim data were released from a phase 2/3 study: a single-arm, open-label clinical trial that assessed the safety, tolerability and pharmacokinetics of RDV in 53 pediatric patients. Children enrolled were at least 28 days of age and weighing at least 3 kg with confirmed SARS-CoV-2 infection and mild, moderate or severe COVID-19 [35]. Patients in this pediatric phase 2/3 trial received RDV for up to 10 days. Adverse events included acute kidney injury (11%) and an increase in alanine transaminase levels (8%). However, this study had no placebo group, thus limiting the possibility to draw conclusions regarding the significance of these adverse events [36].

Interestingly, the analysis of the different variables and the duration of treatment for more or less than 5 days, showed that patients who were administered RDV for more than 5 days waited longer before RDV administration after the detection of COVID-19-associated pneumonia. This might suggest that patient diagnosed with pneumonia need to start antiviral treatment promptly, to avoid getting critically ill.

Most children have milder COVID-19 than adults do, and a small percentage of children require treatment, so few meta-analyses on pediatric treatment have been reported. A systematic review and meta-analysis on the treatment of COVID-19 in children showed that anti-inflammatory agents such as corticosteroids and antivirals such as RDV are the most promising for severe cases of pediatric COVID-19 [37]. In addition, WHO and various guidelines based on large RCTs and systematic reviews suggest that in addition to RDV, corticosteroids, IVIG, tocilizumab, anakinra, infliximab, aspirin, and heparin, other drugs cannot be recommended in cases of COVID-19 in children of any severity [38]. Safety and efficacy data on the use of monoclonal antibodies are reported in the literature [38–41] and show good tolerance and safety, but clear data on efficacy in children are still limited. Ader et al. published on 2022 the results of DisCoVeRy trial showing no clinical benefit from the use of Remdesivir in patients who were admitted to hospital for COVID-19 with symptoms for more than 7 days. However, this trial has been conducted only in adult patient with COVID-19 and highlights that the time of administration, as for other antiviral (e.g. oseltamivir for influenza) might be a key factor for a successful treatment [42]. A recent retrospective study conducted by Manciulli et al. compared the clinical features, 28-day outcomes (hospitalization or death), and drugs tolerability among adult patient treated with sotrovimab (SOT), remdesivir (RMD), nirmatrelvir/ritonavir (NRM/r), or molnupiravir (MOL) [43]. This study showed good safety and efficacy for each drug and highlighted for SOT a reduced risk of progression versus RMD, however no significant differences of outcome were observed in preventing 28-day hospitalization and death among patients treated with RMD, MOL, and NRM/r [43]; no pediatric patients were included in the study.

Our study has some limitations: it is a retrospective descriptive analysis and the results were clinically interpreted. This was because it was not a clinical study designed for data collection. Without comparative data from a randomly assigned control group, it is not possible to say whether the high rate of hospitalizations observed in these patients was due to the effects of RDV, the natural course of the disease, or other therapeutic interventions. Another limitation is the absence of follow-up and SARS-CoV-2 viral load data on nasal swab during treatment: the lack of these data prevents us from assessing the real effect of RDV on virus clearance. However, our results highlight and support the safety and tolerability of RDV administration in pediatric patients, although the absence of a control arm prevents us from determining how much RDV really contributed to recovery. This confirms the need for pediatric clinical trials on the use of RDV for the prevention of severe COVID-19 in high-risk pediatric patients and the need to improve the limited data available on the pharmacokinetics and pharmacodynamics of RDV.

## Conclusion

In conclusion our data suggest that RDV against SARS-CoV-2 is safe and well-tolerated in pediatric populations at high risk of developing severe COVID-19; a delayed administration of RDV therapy after diagnosis of pneumonia may be associated with a longer duration of antiviral therapy, especially in patients with comorbidities.

## Data Availability

All data generated or analyzed during this study are included in this published article.

## Abbreviations

RDV: Remdesivir
ARDS: Acute respiratory distress syndrome
RT-PCR: Real-Time reverse-transcriptase Polymerase-Chain-Reaction
MIS-C: Multisystem inflammatory syndrome temporarily related to COVID-19
SDs: Standard deviations medians
IQRs: Interquartile ranges
ECMO: Extracorporeal membrane oxygenation
ADA: Deficiency, Adenosine deaminase deficiency
CRP: C-reactive protein
ALT: Alanine amino-transferase
AST: Aspartate amino-transferase
